# Education Research: Neurology Residents Report Improved Skills After Initiation of a Lumbar Puncture Clinic

**DOI:** 10.1212/NE9.0000000000200040

**Published:** 2023-01-19

**Authors:** Anna Pfalzer, Heather Koons, Christopher Lee, Lealani Mae Acosta

**Affiliations:** From the Vanderbilt University Medical Center, Nashville, TN.

## Abstract

**Background and Objectives:**

Neurology residents have limited opportunities to perform lumbar punctures (LPs). We hypothesized that establishing a clinic for residents to perform LPs would increase success rates, improve resident comfort with LPs, reduce the need for assistance by attending physicians, and improve patient care.

**Methods:**

The Vanderbilt University Medical Center neurology residency began a resident LP clinic and measured residents' input and clinical data to see whether the clinic affected resident LP skills. Before and after the launch of LP clinic, neurology residents were invited to complete online surveys at the end of the academic year and during their LP clinic rotation. Completion of the surveys was voluntary and considered consent. The surveys assessed LP attitudes and experience (e.g., confidence with LPs and number performed) and LP clinic procedural data (e.g., LP success rate). Attitudes were measured by assessing confidence; experience by quantifying the number of LPs performed; procedural success was measured by the number of LPs with successful CSF acquisition. Differences in resident attitude and LP outcomes were analyzed using Spearman correlations and logistic regressions.

**Results:**

Prior to the launch, 15/25 (60% response) residents responded to the clinic survey. After the launch, 6/21 (29%) responded to the first-year follow-up survey and 12/21 (57%) to the second-year follow-up survey. Resident confidence and the number of LPs performed were unchanged. Success rate reported by individual residents increased 15% (*p* = 0.04), which did not correlate with the overall LP clinic success rate. In the first year of the clinic, 83% of postgraduate year (PGY)3s needed an attending's assistance compared with 29% of PGY4s. In the second year, 44% of PGY3s and 32% of PGY4s needed an attending's assistance.

**Discussion:**

This structured clinic provided an opportunity for informal resident-to-resident teaching, which may have reduced the need for attending assistance.

Physicians, particularly neurologists, need to be trained to perform diagnostic and therapeutic lumbar punctures (LPs) during residency.^1^ Although the number of LPs a resident performs does not equate skill, substantial exposure is necessary.^2^ Residents perform LPs in various scenarios, such as emergent diagnoses, such as CNS infection or suspected intracranial hypertension, or with routine evaluation, such as for neuroinflammatory disorders.^3^

Providing a supportive environment with adequate opportunities to master LP skills is critical in physician training. Residents frequently learn from more senior residents how to perform an LP. Previous data regarding LPs demonstrate that multiple factors may lead to an unsuccessful LP (e.g., high body mass index [BMI]).^4,5^ Such experiences may discourage physicians-in-training as they develop proficiency.

Because there was no formal curriculum for training with LPs, the Vanderbilt University Medical Center (VUMC) neurology residency training program developed an outpatient LP clinic. Patients were referred as part of a routine diagnostic and/or therapeutic intervention. We hypothesized that a structured LP clinic will improve resident attitudes and procedural success.

## Methods

This study was an observational cohort study to assess the impact of an LP clinic on VUMC neurology resident skills and success performing LPs. VUMC pediatric and adult neurology residents are typically in their postgraduate year (PGY)3 or PGY4 and rotate weekly through LP clinic, with each resident rotating through several times a year. Each resident completed surveys administered through Research electronic data capture (REDCap),^6,7^ a secure web-based software platform designed to support data capture for research studies. Residents completed an anonymous survey, the Resident Perception survey (eAppendix 1, links.lww.com/NE9/A13), about resident LP experience between May 2018 and June 2018 (prior to the launch of the LP clinic in June 2018) and annually thereafter. This study examined resident responses from the first 2 years of the LP clinic between June 2018 and June 2020. We pooled data from these 2 years because of the small sample size and because there was no significant difference in outcome variables because there was not a significant difference in attitudes, number of LPs, and success rate in the following 2 years that the annual survey was distributed after the launch of LP clinic. The Resident Perception survey contained 6 questions about their experience completing LPs. Residents indicated their PGY status, estimated number of LPs performed during residency, estimated number of LPs performed during the current PGY and cumulative over residency to date, comfort (5-item Likert scale), confidence level, and success rate (both on a sliding scale from 0% to 100%).

The second survey, the Clinical Outcomes survey (eAppendix 2, links.lww.com/NE9/A14), was for each LP performed in LP clinic, in which the residents filled out a REDCap form regarding patient demographics (age, weight, height, and BMI, which was also subdivided into overweight [BMI >25 kg/m^2^] and obese [BMI >30 kg/m^2^]), PGY level, estimated number of cumulative LPs performed during residency, indications for patient to get an LP, whether it was “successful” (as defined by acquiring the CSF), and whether the attending performed part of the LP.

Owing to anonymity, we are unable to validate resident responses and unable to identify individual residents who completed the survey pre-LP clinic and post-LP clinic. This limitation prevented the assessment of change in assessments prelaunch and postlaunch. All residents were eligible for this study, and no responses were excluded from these analyses.

### Standard Protocol Approvals, Registrations, and Patient Consents

IRB approval (#201701) was obtained within VUMC's ethical standards committee. Because the LPs were part of routine clinical care, written consent was not necessary from the patients. Residents did not complete a formal consent form because responses were anonymous. Voluntary participation was considered consent. We used the SQUIRE checklist when writing our report.^8^

### Statistical Analysis

The effect of the LP clinic on resident attitudes, experience with LPs, and success rate was examined using logistic regression models comparing responses from preclinic to postclinic. Correlations between outcome measures were assessed using Spearman correlations. Differences in perceived confidence and success rate were assessed using a Mann-Whitney test. All analyses were performed on GraphPad Prism (version 9.2). Categorical variables were analyzed using binary or multivariate models (logistic regression vs multiple logistic regression) and reported as frequency (%). Continuous variables were reported as individual incidences (n) and mean ± SD. Statistical significance was set at *p* < 0.05.

### Data Availability

Anonymized data not published within this article will be made available by request from any qualified investigator.

## Results

Participant responses to the Resident Perception survey (eAppendix 1, links.lww.com/NE9/A13) and Clinical Outcomes survey (eAppendix 2, links.lww.com/NE9/A14) are outlined below in Tables 1 and 2, respectively.

**Table 1:** Resident Perception Survey: Participant Demographics Pre-LP and Post-LP Clinic Establishment (eAppendix 1, links.lww.com/NE9/A13)

Variables	Preclinic	Postclinic		
	2018	2018–2019	2019–2020	2018–2020
PGY, n (%)				
PGY2	5 (33)	2 (33)	3 (25)	5 (28)
PGY3	6 (40)	2 (33)	5 (42)	7 (39)
PGY4	4 (27)	2 (33)	4 (33)	6 (33)
Total	15 (100)	6 (100)	12 (100)	18 (100)
Confidence, mean ± SD				
PGY2	62.0 ± 18.4	60.0 ± 14.1	53.0 ± 29.6	55.3 ± 24.1
PGY3	62.8 ± 28.2	77.0 ± 2.8	72.2 ± 14.3	73.5 ± 11.9
PGY4	81.3 ± 2.5	100.0 ± 0.00	69.3 ± 13.3	79.5 ± 18.9
Total	65.4 ± 20.2	79.0 ± 19.1	69.7 ± 20.4	71.9 ± 20.1
Success rate, mean ± SD				
PGY2	69.4 ± 20.4	57.0 ± 9.8	78.0 ± 10.2	71.0 ± 14.1
PGY3	55.6 ± 18.1	82.5 ± 3.5	73.4 ± 7.2	76.0 ± 7.5
PGY4	72.7 ± 1.7	87.0 ± 5.7	73.8 ± 7.6	78.1 ± 9.4
Total	65.2 ± 17.0	75.5 ± 15.4	74.9 ± 7.9	75.11 ± 10.4^a^

Abbreviations: LP = lumbar puncture; PGY = postgraduate year.

Table 1 outlines the breakdown in responses by PGY and preclinic (2018) or postclinic (2018–2020) establishment. Preclinic and postclinic comparisons were analyzed using the gray highlighted columns (2018 vs 2018–2020).

a Statistical significance at *p* < 0.05 compared with preclinic success rate.

**Table 2:** Clinical Outcomes Survey: Participant Demographics Pre-LP and Post-LP Clinic Establishment (eAppendix 2, links.lww.com/NE9/A14)

Variable	Preclinic	Postclinic		
	2018	2018–2019	2019–2020	2018–2020
Attending assistance (2018–2019, 2019–2020)				
PGY2 (1, 2)	N/A	1 (100%)	1 (50%)	2 (67%)
PGY3 (6, 7)	N/A	5 (83%)^a^	2 (29%)	7 (54%)
PGY4 (13, 17)	N/A	3 (23%)	8 (47%)	11 (37%)
Total	N/A	9 (45%)	11 (42%)	20 (43%)

Abbreviations: LP = lumbar puncture; N/A = not available; PGY = postgraduate year.

A greater number of residents completed separate surveys relating to LP completion; thus, sample size for the variable “attending assistance” is shown separately for that variable. For attending assistance, percentages reflect the percent by PGY.

a Statistical significance at *p* < 0.05 compared with PGY4s.

For the Resident Perception survey, 15 of 25 (60% response rate) residents completed the survey related to their experience prior to the launch of LP clinic (preclinic). Six residents of 21 (29% response rate) completed surveys at the end of the first year the clinic was established (established in 2018, surveys completed in 2019), while 12 of 21 (57% response rate) residents completed the surveys the following year (2020). Of importance, there were no differences in resident perception responses (i.e., number of LPs and confidence level) for both years postclinic; thus, responses were combined as “postclinic.”

The response rate for the Clinical Outcomes survey regarding LP procedural success included 1 PGY2, 6 PGY3s, and 13 PGY4s in 2018–2019 and 2 PGY2s, 7 PGY3s, and 17 PGY4s in 2019–2020, as summarized in Table 2.

From the Clinical Outcomes data, of the 60 LPs performed, 19 patients (31.7%) were undergoing the procedure as part of a neurodegenerative dementia process, such as Alzheimer disease, 5 (8.3%) were being worked up for normal pressure hydrocephalus, 8 (13.3%) were for idiopathic intracranial hypertension, 12 (20%) were for an autoimmune disease (e.g., multiple sclerosis), 1 (1.7%) was for an infectious etiology, and 15 (25%) were for other indications.

### Survey: Resident Perception

**Figure 1:**
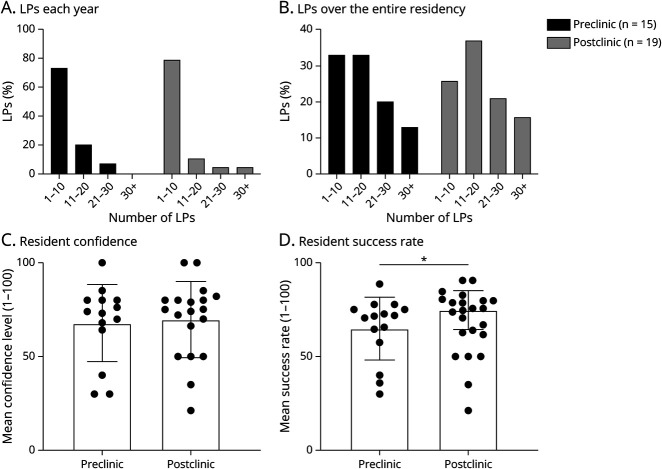
Participant Responses to Perception Survey Regarding (A) Estimated Number of Lumbar Punctures (LPs) Completed Annually, (B) Estimated Number of LPs Completed Over the Course of Residency, (C) Participant Perceived Confidence in Completing LPs and (D) Participant Perceived Success Rate Preclinic and Postclinic Establishment Data for (A) and (B) are shown as percentage with statistical differences determined using logistic regression. Data for (C) and (D) are shown as mean ± SD with statistical significance set at *p* < 0.05 and determined using Mann-Whitney tests.

Similarly, we found no difference in the level of resident confidence in performing LPs between preclinic and postclinic (Figure 1C; Table 1, *p* = 0.75); however, resident perceived success rate increased 15% postclinic compared with preclinic (Figure 1D; Table 1, *p* = 0.04). LP clinic establishment did not change the estimated number of LPs completed by residents per year or over the course of residency (Figure 1, A and B). Specifically, preclinic, 73% of residents completed 1–10 LPs, 20% completed 11–20 LPs, and 7% completed 21–30 LPs annually during residency. Postclinic, 79% of residents completed 1–10 LPs, 11% completed 11–20 LPs, 5% completed 21–30 LPs, and 5% completed more than 30 LPs annually during residency.

### Survey: Clinical Outcomes

As summarized in Table 2, in the first year of LP clinic, 83% of PGY3s required assistance by an attending compared with 23% of PGY4s: PGY3s were 13 times more likely to need assistance than PGY4s (odds ratio [OR] 13.21, 95% CI 2.14–40.2; *p* = 0.03). After the second year of LP clinic, PGY3s were as likely to need assistance as PGY4s (29% vs 47%) (OR 0.97; 95% CI 0.2892–3.212; *p* = 0.97).

**Figure 2:**
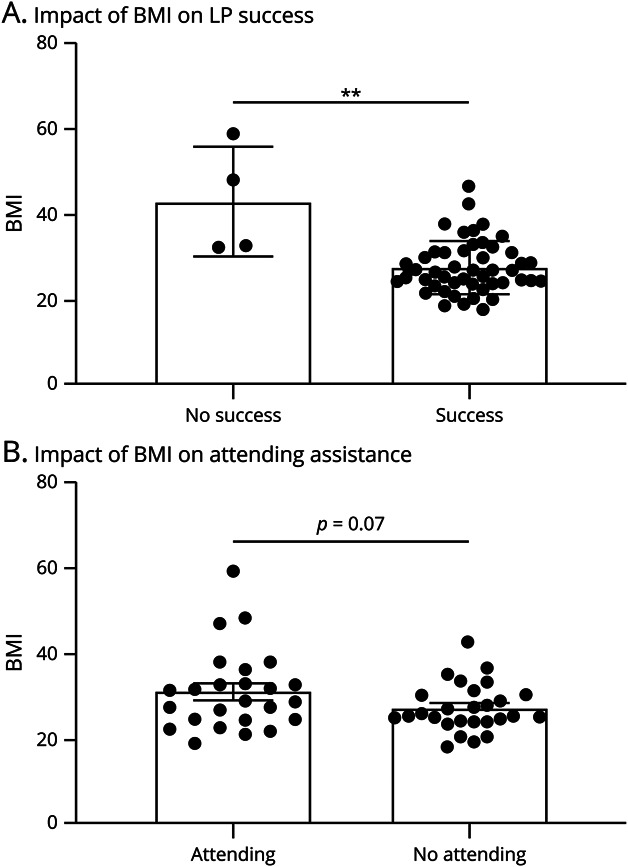
Role of Patient BMI on Neurology Residents (A) Successful Completion of LP and (B) Need for Assistance by the Attending Physician Data are reported as mean ± SD, and an asterisk (**) indicates *p* < 0.01 between groups using a Mann-Whitney test. BMI = body mass index; LP = lumbar puncture.

The bedside success rate in the first year of LP clinic was 95.2% and in the second year was 92.3%. Only 4 LPs attempted in the LP clinic were unsuccessful (no collection of CSF): those attempts were with patients with higher BMIs (Figure 2A; *p* = 0.005). Patients with higher BMIs tended to require attending assistance compared to those patients with lower BMIs (Figure 2B). Specifically, obese patients were 70% more likely to need attending assistance (*p* = 0.05).

## Discussion

The increased exposure and training to develop proficiency with LPs during residency is likely beneficial for residents and patients. After LP clinic inception, we identified improvements in reported resident success, though not in confidence, and a decreased need for PGY3 residents to require attending assistance in the first year after LP clinic launch, though this was not the case in the second year of clinic.

Our establishment of a weekly LP clinic to provide neurology residents with additional structured training is important because limited studies exist that monitored outcomes launching such a clinic.^9^ Our findings may serve useful for other residencies who want to build a similar clinic to improve procedural training, which is likely feasible for many programs. This weekly clinic is supervised by an attending who has experience with hundreds of clinical and research LPs and whose schedule allows for being at the bedside from needle in to needle out while the resident performs the LP. Primarily, PGY3 and PGY4 residents rotate through the clinic, though a PGY2 may elect to come to clinic for additional LP experience. Two LPs are performed per clinic: 1 LP as part of an NPH work-up, with pre-LP and post-LP assessments, and 1 non-NPH LP for another clinical indication (e.g., dementia, demyelinating disease). The structure of the clinic has been conducive to resident education, with many of them expressing appreciation for the tips and guidance provided by the experienced attending in addition to the attending being back-up if the resident is unable to acquire the CSF, with an excellent success rate.

Such a clinic can assist neurologists in the work-up for a variety of indications while giving residents personalized guidance in the procedure. Specific results, such as BMI data, may influence guidelines for clinic LPs. For example, we are considering screening BMI to determine whether obese patients are instead referred for a fluoroscopically guided LP, to minimize patient discomfort and the number of attempts to get an LP. The residents have emphasized the importance of having a seasoned attending with years of experience performing LPs as critical to a positive learning environment. When physicians place an order for an LP to be performed in this clinic, they must specify certain information to ensure the LP is performed safely and properly, such as whether the patient is on anticoagulation that needs to be held, how much CSF they want removed, and what labs are to be ordered. Nursing verifies the orders ahead of the scheduled visit to ensure the necessary details are in order to proceed with LP.

Our study has several limitations. Our correlation does not necessarily equate causation, though, because this may reflect an expected maturation effect of the residents as they progress through residency. Other variables, such as the LPs performed outside of clinic, may also have affected responses. The number of participants is small, and this sample is chosen out of convenience as opposed to random selection. It is also possible that responses are biased toward those residents with more favorable responses and experiences toward the LP clinic. The preclinic and postclinic resident surveys were anonymous and subjective, so individual change could not be monitored and subjective recall of the residents (e.g., number of LPs performed) independently verified. The change in the percentage of PGY3s compared with PGY4s needing assistance could be a practice effect because the previous year's PGY3s responded to the next year's survey as PGY4s. Because other variables did not change, such as the documented success rate for LP clinic LPs, this does not appear to be a practice effect. Our hypothesis is that LP clinic gave residents more practice to improve their independence, putting PGY3s and PGY4s on equal footing with their need for attending intervention. Previous studies have in fact demonstrated that LP simulators that can mimic certain clinical conditions do improve LP skills in trainees.^5,10^ It is unknown whether residents independently practiced on such simulators outside of the LP clinic.

Launching an LP clinic improved residents' perceived success rate and independence in performing LPs without attending assistance. We will continue to collect and analyze data to determine variables affecting resident LP skills.

Establishing a neurology LP clinic improved residents' reported success performing LPs by 15%, though did not affect confidence or estimated number of LPs performed. Initially, PGY3s were 13 times more likely to need assistance compared with PGY4s, a difference that did not remain after LP clinic had been established at least 1 year.

## Appendix Authors

**Table:**

Name	Location	Contribution
Anna Pfalzer, PhD	Vanderbilt University Medical Center, Nashville, TN	Drafting/revision of the article for content, including medical writing for content; analysis or interpretation of data
Heather Koons, MD, MPH	Vanderbilt University Medical Center, Nashville, TN	Drafting/revision of the article for content, including medical writing for content; major role in the acquisition of data; and study concept or design
Christopher Lee, MD, MPH	Vanderbilt University Medical Center, Nashville, TN	Drafting/revision of the article for content, including medical writing for content; major role in the acquisition of data; and study concept or design
Lealani Mae Acosta, MD, MPH	Vanderbilt University Medical Center, Nashville, TN	Drafting/revision of the article for content, including medical writing for content; major role in the acquisition of data; study concept or design; and analysis or interpretation of data

## Glossary

BMI: body mass index
LP: lumbar puncture
OR: odds ratio
PGY: postgraduate year
REDCap: research electronic data capture
VUMC: Vanderbilt University Medical Center
